# Multiple facets in the control of acromegaly

**DOI:** 10.1007/s11102-013-0536-7

**Published:** 2013-11-23

**Authors:** Lucio Vilar, Alex Valenzuela, Antônio Ribeiro-Oliveira, Claudia M. Gómez Giraldo, Doly Pantoja, Marcello D. Bronstein

**Affiliations:** 1 Division of Endocrinology and Metabolism, Hospital das Clinicas, Federal University Medical School, Recife, Pernambuco Brazil; 2 Department of Internal Medicine, Fundación Cardio-Infantil, Instituto de Cardiología, Universidad del Rosario, Bogotá, Colombia; 3 Department of Internal Medicine, Federal University of Minas Gerais, Belo Horizonte, Minas Gerais Brazil; 4 Hospital Universitario de San Ignacio and Organización Colsánitas Internacional, Bogotá, Colombia; 5 Universidad de Nariño, Pasto, Colombia; 6 Neuroendocrine Unit, Division of Endocrinology and Metabolism, Hospital das Clinicas, University of São Paulo Medical School, Av. Dr. Eneas de Carvalho, 255, 7ºandar, sala 7037, São Paulo, CEP 05403-000 Brazil

**Keywords:** Acromegaly, Pituitary, Latin America, GH, IGF-1, Tumor shrinkage, Comorbidities, Somatostatin analogs

## Abstract

**Aims:**

The current article provides a brief overview of the criteria for defining disease control in acromegaly.

**Methods:**

This was a retrospective, narrative review of previously published evidence chosen at the author’s discretion along with an illustrative case study from Latin America.

**Findings and Conclusions:**

In the strictest sense, “cure” in acromegaly is defined as complete restoration of normal pulsatile growth hormone secretion, although this is rarely achieved. Rather than “cure”, as such, it is more appropriate to refer to disease control and remission, which is defined mainly in terms of specific biochemical targets (for growth hormone and insulin-like growth factor-1) that predict or correlate with symptoms, comorbidities and mortality. However, optimal management of acromegaly goes beyond biochemical control to include control of tumour growth (which may be independent of biochemical control) and comprehensive management of the symptoms and comorbidities typically associated with the disease, as these may not be adequately managed with acromegaly-specific therapy alone.

## Introduction

Acromegaly is a disease of excessive growth hormone (GH) secretion and the primary aims of treatment are to control GH secretion or its effects on GH-sensitive tissues, most notably increased insulin-like growth factor-1 (IGF-1) secretion [1]. Intrinsically, “cure” in acromegaly should be characterized by complete restoration of normal pulsatile GH secretion, but this is rarely achieved [2]. Thus, it is more appropriate to refer to disease control and remission, which in turn is defined based on specific GH and IGF-1 targets that predict or correlate with symptoms, co-morbidities and mortality [3]. Tumour mass reduction or disappearance is also used in establishing disease remission or control [3]. In surgically treated patients, remission is defined by both the normalization of age-adjusted IGF-1 serum concentrations, a random serum GH below 1 μg/L and/or a glucose-suppressed GH level below 0.4 μg/L. In patients treated with SSAs and/or dopamine agonists, adequate control is defined by the achievement of a normal IGF-1 concentration and a “safe” GH level (see below). In this context, a “safe” GH level alludes to a concentration of the hormone below which the increased mortality is reduced to that seen in the general population [3–6]. However, optimal management of patients with acromegaly extends beyond biochemical control to include factors such as tumor growth and attention to comorbid conditions. The current article summarizes the criteria for defining disease control in acromegaly, with an illustrative case study and a particular focus on issues in Latin America.

**Fig. 1:**
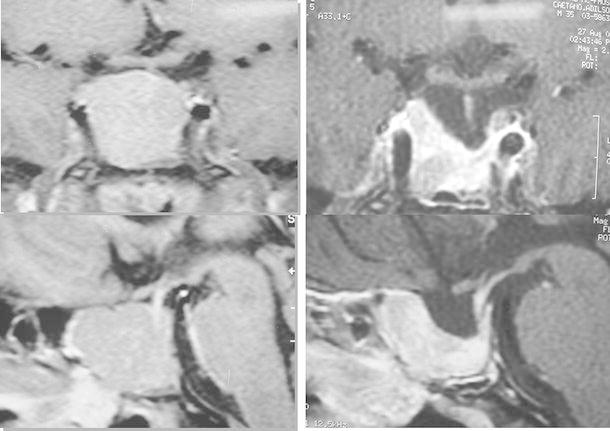
MRI scans of pituitary tumor at diagnosis (April 2008, left) and following non-curative surgery (January 2009, right) prior to commencing pharmacological therapy

## Measurement of GH and IGF-1 in acromegaly

Assays for GH and IGF-1 have evolved considerably over the past two decades, from the less specific and sensitive radioimmunoassays (RIAs), to a myriad of commercially available ultrasensitive immunoassays that use more specific monoclonal antibodies, which are capable of detecting much lower hormone concentrations [7, 8]. Several analytical and physiological issues need to be taken into account when using GH and IGF-1 assays in the diagnosis and follow-up of patients with acromegaly. This includes the standard preparation used (the so-called International Reference Preparation or “IRP”), which at present should be the recombinant World Health Organization (WHO) second 95/574 preparation for GH and the recombinant WHO second 02/254 preparation for IGF-1 [7–10]. Other analytical aspects to be considered are the specificities of the monoclonal antibodies and, in the case of IGF-1, the interference from IGF-1-binding proteins [9].

Several physiological and pathological states or conditions influence GH and IGF-1 synthesis and secretion. For instance, GH is secreted in pulses occurring mainly at night; thus, a random measurement is not useful, except when the result is particularly low (less than 0.4 μg/L), which confidently excludes the diagnosis [3]. Furthermore, IGF-1 concentrations decrease with age, reflecting the parallel decline of the somatotropic axis, and thus need to be adjusted accordingly. Ethnogenetic factors may also influence IGF-1 levels and, ideally, normal ranges should be established locally using serum from a significant number of age-stratified healthy individuals [9, 11]. In addition, several conditions can lower IGF-1 levels, including malnutrition, poorly controlled diabetes, and hepatic or renal failure, as well as estrogen therapy and hypothyroidism [9, 11].

## Biochemical versus symptom control

Based on meta-analyses, mortality of treated acromegaly patients whose serum GH level is below 2.5 μg/L (measured by RIA) is similar to that seen in the general population [12, 13]. In contrast, a serum GH level greater than 2.5 μg/L confers an increased risk of mortality [12, 13]. A similar pattern is seen for IGF-1 when comparing normal age-adjusted levels versus levels above the age-adjusted normal range [12, 13]. Based on these observations, clinicians treating acromegalic patients are advised to aim for random serum GH <2.5 μg/L measured by RIA (probably <1 μg/L measured by modern sensitive immunoassay) and normal IGF-1 levels, and this represents the current definition of controlled disease or “cure” [2, 3, 12, 13].

Unfortunately, there are patients who meet the above criteria for GH who are still symptomatic, have clear evidence of progression of co-morbidities or who have abnormally elevated IGF-1 [2, 14]. Thus, measured values of IGF-1 and random GH may yield a discrepant prediction of disease stabilization, especially in terms of symptomatic disease [2, 14]. In these cases, the IGF-1 level may provide a better measure of average GH secretion, as it correlates well with signs and symptoms of active disease, such as soft-tissue thickening and insulin insensitivity [2, 14, 15]. Furthermore, IGF-1 seems to be a better predictor of disease control than random GH [2, 15]. Although cumbersome and less practical, whenever random GH values are discrepant, multiple GH sampling during a 2-h period with a mean value <1 μg/L may be used to indicate adequate disease control [16].

Suppression of GH during an oral glucose tolerance test (OGTT) is of limited value in evaluating disease control in many patients, being helpful only in those who are not receiving any pharmacological treatment, most notably in the postoperative setting [2, 3, 16, 17]. Some evidence also suggests that radiotherapy can exaggerate the discordance between disease activity assessed by IGF-1 and GH suppression during an OGTT (if GH nadir is <2.5 μg/L) [18]. In treatment-naïve, biochemically active patients, discordance appears to be greatest in those with only mildly elevated GH output, leading to a high false negative rate with GH suppression measurement [19]. Increased discordance is also seen at the other extreme in patients with particularly high GH levels [20]. In patients treated with pegvisomant, only IGF-1 remains a reliable marker of disease activity, as GH concentrations remain elevated [3, 16].

Thus, for patients receiving medical treatment with SSAs or dopamine agonists, IGF-1 and random GH measurements together are sufficient for assessment of biochemical response [2, 3]. The IGF-1 level, in particular, can be an important determinant of the need for additional therapy, although results can be influenced by the presence of malnutrition, poorly controlled diabetes mellitus, hypothyroidism, liver function impairment, renal failure, inflammatory diseases and malignancies [9, 11, 21]. Finally, it should be noted that the effects of pharmacological therapies on tumor size may not necessarily be related to biochemical remission, and this may represent an aspect of disease control that deserves separate consideration [3, 22–24].

## Tumor shrinkage

Tumor size reduction is an important goal in the management of acromegaly [3, 6]. In the first-line clinical setting, control of both GH secretory activity and tumor growth are required in order to achieve comprehensive therapeutic efficacy [3, 6]. The clinical relevance of tumor shrinkage may be greater in macroadenomas than in microadenomas [24]. The clinical benefits of tumor mass shrinkage include relief of optic chiasm impingement, patient reassurance that the mass is shrinking, and possibly a lowered risk of intratumoral hemorrhage [25]. Such effects have the potential to provide a noticeable beneficial impact on patient quality of life.

In addition to the achievement of biochemical control, some pharmacological therapies can also induce significant tumor shrinkage in patients with acromegaly [24–28]. Tumor shrinkage has been reported in approximately two-thirds of patients treated with long-acting SSAs and one-third of patients treated with dopamine agonists [24, 28]. The SSAs being used today are almost exclusively longer-acting, depot formulations and they seem to provide more benefit in reducing tumor size than the old shorter-acting formulations [24, 29]. The effects on tumor size are especially marked when these agents are used as first-line therapy [29]. In a recent study involving 30 newly diagnosed unselected patients receiving SSA therapy for 24 weeks, 97 % experienced a reduction in tumor volume, 79 % had a reduction ≥20 % and the median reduction in volume was 39 % [30]. In the case of SSAs, the anti-tumor effects may relate, at least in part, to direct effects on cell growth and indirect effects via inhibition of angiogenesis [27]. The available evidence suggests that pegvisomant does not reduce tumor size, at least when used as monotherapy [31]. Earlier concerns regarding pituitary adenoma growth during pegvisomant therapy now seem to have settled to an estimated risk of about 3 % thanks to long-term follow-up studies [32].

## Control of comorbidities

Major comorbidities associated with acromegaly include cardiovascular disease (including cardiomyopathy), diabetes, hypertension, sleep apnea, and arthritis [3, 33–36]. Acromegaly is also associated with a greater risk of several neoplasms, particularly colonic polyps and carcinoma, and there is growing evidence that the risk of thyroid tumors is also increased [33, 37–40]. Furthermore, in addition to hypertension and diabetes, active acromegaly is associated with several other classic and nonclassic cardiovascular risk factors, including insulin resistance and dyslipidemia, as well as increased levels of fibrinogen and lipoprotein (a) [41].

Biochemical control has been shown to provide improvements in several comorbidities of acromegaly, especially cardiomyopathy, sleep apnea, and arthralgia, but also hypertension and dyslipidemia [3, 42, 43]. In one study involving 30 patients with newly diagnosed acromegaly, 12 months of SSA therapy decreased joint thickness in all cases, but the reduction was greater in those with controlled disease, among whom 61 % had normalization of shoulder thickening and 89 % had normalization of knee thickening [33]. Similarly, successful biochemical control after 12 months of SSA therapy has been shown to normalize left ventricular (LV) hypertrophy in 100 % and LV ejection fraction in 80 % of patients under 40 years of age (but only 50 % of patients over 40 years of age for either measure) [33]. These results are supported by a meta-analysis of 18 SSA trials, which found a significant reduction in LV mass and several functional hemodynamic parameters [44]. In a recent study, LV mass regression was reported in men (but not women) and there were also significant improvements in arterial stiffness and endothelial function after 24 weeks of SSA therapy [30]. In the same study, 61 % of the 30 patients exhibited an improvement in sleep apnea, but 30 % experienced worsening and 9 % had no change [30]. Thus, effective biochemical control does not always result in effective control of these comorbidities and improvements may be limited, in spite of normalized GH levels [3, 41, 42, 45]. Additional therapies are, therefore, frequently required to treat comorbid conditions in acromegaly. In particular, effective control of diabetes, hypertension and dyslipidemia is essential in order to reduce the increased vascular morbidity and mortality associated with these key cardiovascular risk factors [3, 42]. Fortunately, good glycemic control can be achieved in the majority of acromegalic patients with type 2 diabetes using standard approaches, such as lifestyle intervention, oral glucose-lowering agents and insulin [46]. Hypertension in acromegaly is also easily controlled with standard antihypertensive medications [47]. Regarding dyslipidemia, statin therapy has been shown to provide significant improvements in atherogenic lipid profile and reduce calculated coronary heart disease risk in patients with acromegaly [48].

**Table:** Case study: Addressing multiple comorbidities in a patient with acromegaly (Lucio Vilar, MD, PhD)

*A 40* *year-old female was referred to the endocrinologist in April 2008 due to amenorrhea over the previous 10* *months*
Symptoms
Increased shoe size (from 35 to 38)
Oily skin, excessive sweating
Excessive snoring
Amenorrhea (10 months)
Polyarthralgia
Signs
Height: 1.56 m
Weight: 66.3 kg
Blood Pressure (BP): 160/100 mmHg
Enlarged hands and feet
Macroglossia, diastema
Prognathism, dental malocclusion
No goiter
Personal and family history
No family history of diabetes, cancer, thyroid disease or pituitary disease
The last medical evaluation was made in 2004; no biochemical abnormality was found
Lab tests
GH (ICMA): 23.8 μg/L
IGF-1 (ICMA): 960 μg/L (normal 101–267 μg/L)
GH nadir during OGTT: 6.3 μg/L
Prolactin and thyroid function tests: normal
Estradiol: 46 pmol/L (12.6 pg/mL)
FSH: 0.8 IU/L
Fasting plasma glucose: 7.6 mmol/L (137 mg/dL)
HbA_1c_ = 7.4 %
Serum calcium: normal
Triglycerides: 5.5 mmol/L (487 mg/dL)
HDL cholesterol: 0.8 mmol/L (31 mg/dL)
Diagnosis
Acromegaly caused by a GH-secreting pituitary macroadenoma
MRI
Macroadenoma (2.3 × 1.8 cm), with infrasellar, parasellar and suprasellar extension (Fig. 1)
Computerized visual field testing ⇒ Normal
Echocardiogram
Marked left ventricular (LV) hypertrophy
No valvular abnormalities
Treatment
Patient was submitted to transsphenoidal surgery in Sept 2008, which was not curative (Fig. 1)
IGF-1: 802 μg/L (normal 101–267 μg/L)
GH: 13.6 μg/L
GH nadir: 3.7 μg/L
SSA was started in March 2009, followed by a higher dose in May 2009 followed by SSA + cabergoline 3 mg/week
IGF-1 response to medical treatment showed improvement to normal range (101–267 μg/L) with sequential medical therapy from 780 μg/L to 253 μg/L
Thyroid ultrasound
June 2008: normal
January 2011: 1.7 cm hypoechoic solid nodule with increased blood flow in right lobe
FNA biopsy ⇒ Papillary thyroid carcinoma
March 2011 ⇒ Total thyroidectomy
Thyroid histology ⇒ *BRAF* ^V600E^-positive tall cell variant papillary thyroid carcinoma
Patient’s comorbidities
Thyroid carcinoma
Diabetes mellitus
Dyslipidemia
Left ventricular hypertrophy
Polyarthralgias
Central hypogonadism
Excessive snoring (sleep apnea?)
Effect of treatment on comorbidities
Thyroid carcinoma ⇒ No evidence of disease recurrence or metastasis after total thyroidectomy and ^131^I abalation therapy
Diabetes ⇒ Metformin XR (750 mg/d) required to control blood glucose and HbA_1c_ levels
Dyslipidemia ⇒ resolved with the improvement of diabetes and normalization of GH and IGF-1 levels
Hypertension ⇒ BP control achieved with losartan + amlodipine + indapamide
Central hypogonadism/excessive snoring ⇒ resolved with GH/IGF-1 normalization
LV hypertrophy ⇒ improvement after BP control and hormonal normalization

## Case discussion

This case provides a good example of a patient with a burden of multiple comorbidities typical of acromegaly, including diabetes, dyslipidemia and hypertension (all of which are major cardiovascular risk factors), cardiomyopathy, arthralgia, and sleep apnea, along with thyroid carcinoma. After surgical failure, biochemical control and tumor shrinkage was achieved through the use of pharmacological therapy, which was ultimately successful using a combination of SSA and dopamine agonist [49, 50]. Biochemical control was associated with improvements in some comorbid conditions (e.g., excessive snoring/sleep apnea) and may have contributed to amelioration of dyslipidemia and cardiomyopathy. However, appropriate specific therapies for diabetes (metformin), hypertension (losartan/amlodipine/indapamide) and thyroid carcinoma (thyroidectomy/^131^I therapy) were required to provide complete management of acromegaly and its comorbidities. At present, blood pressure and diabetes remain well controlled and the patient has not had recurrence of thyroid carcinoma.

## Conclusions

If managed appropriately, most patients with acromegaly should be able to achieve disease control without excess morbidity or mortality, although success may be limited by the modes of treatment and specific drugs available to the treating physicians [3]. The main criteria for “disease control” (rather than the more impracticable concept of “cure”) involve achievement of pre-defined targets for GH and IGF-1. These are based on the levels of GH and IGF-1 that have been shown to be associated with improved symptoms and reduced frequency and severity of comorbid conditions, as well as mortality levels approaching those of the general population [3, 6]. However, beyond these biochemical targets, other factors are also important goals in the management of patients with acromegaly, such as reduction in tumor size (which can be achieved in the majority of patients receiving long-acting SSAs and, to a lesser extent, with dopamine agonists), and more targeted control of comorbid conditions [3, 6]. While some comorbid conditions may be improved to a limited degree with acromegaly-specific therapies alone, they often require other more specific therapies for comorbidities, including the use of antihypertensive, antihyperglycemic and lipid-modifying drugs to control diabetes and reduce the risk of cardiovascular disease. Thus, optimal management of acromegaly encompasses biochemical control, tumor growth control and comprehensive management of the comorbidities commonly associated with acromegaly, such as diabetes mellitus, hypertension and dyslipidemia, which generally respond well to standard therapy .
